# Evaluation of Nutritional Composition of The Dried Seaweed *Ulva lactuca* from Pameungpeuk Waters, Indonesia

**DOI:** 10.21315/tlsr2017.28.2.9

**Published:** 2017-07-31

**Authors:** Abdullah Rasyid

**Affiliations:** Research Center for Oceanography, Indonesian Institute of Sciences, Jl. Pasir Putih No. 1 Ancol Timur, Jakarta 14430, Indonesia

**Keywords:** Seaweed, *Ulva lactuca*, Nutritional Composition, Indonesia

## Abstract

The nutritional composition of the dried seaweed *Ulva lactuca* from Pameungpeuk waters, including proximate, vitamins, minerals, dietary fibre and heavy metal has been carried out. The objective of this present study is to know the nutritional composition of the dried seaweed *U. lactuca* for utilisation in human nutrition in the future. Results show that carbohydrate was the major component in the proximate analysis of *U. lactuca* in the present study. The carbohydrate content was 58.1%. Moisture, ash, protein and fat content were 16.9%, 11.2%, 13.6% and 0.19% respectively, while dietary fibre was 28.4%. The vitamin A content was examined in this study less than 0.5 IU/100 mg while vitamin B1 (thiamine) and vitamin B2 (riboflavin) were 4.87 mg/kg and 0.86 mg/kg respectively. The calcium content was 1828 mg/100 g higher than other minerals. The heavy metal content examined in this study were lower than the limit of the quality criteria applied to edible seaweeds sold in Indonesia. Based on the results of this study show that *U. lactuca* has potential to be developed as an alternative source of a healthy food for human in the future.

## INTRODUCTION

Many current and potential uses of seaweeds have been identified and these have been separated into 10 categories: (1) agriculture, horticulture and agronomy, (2) uses in animal aquaculture, (3) aesthetics, (4) cosmetics, (5) environmental health, monitoring and remediation, (6) food, (7) health, thalassic and wellness, (8) industry, (9) pharmaceutical and pharmacology and (10) science, technology and biomedicine (Apaydin *et al.* 2010).

Seaweeds have been used as food, animal feeds, fertiliser and as sources of traditional medicine in many Asian civilisations since ancient times. Seaweeds are excellent dietary sources of vitamins, proteins, carbohydrates and trace elements. In order to fully exploit the nutritional composition of various seaweeds collected from different part of the world have conducted (Rohani-Ghadikolael *et al.* 2012). The biochemical composition of marine seaweeds is generally known to be highly influenced by geographical location and local environmental condition (Rohani-Ghadikolalel *et al.* 2012).

Seaweed not only possess nutrient potentials, but also nutraceutical potentials like antioxidant, antimutagenic, anticoagulant, anticancerous and antibacterial activity (Abirami & Kowsalya 2011). Hence, seaweeds can be considered as promising plants forming one of the important marine living resources of high nutritional value. Being plants of unique morphology and biochemical composition, *U. lactuca* could be exploited for their multifunctional properties in the form food, energy, medicine and as biotechnological tools (Abirami & Kowsalya 2011).

Since prehistoric time, seaweeds had been remained a staple and vital part in Chinese, Japanese and Korean diet. Interestingly, China and Japan are the main contributors in world production-consumption scenario. Twenty percent of Asian diet is comprised of seaweeds that are relished not for their nutritional viewpoint but of unique and enchanting flavour. But in Western diet, the seaweeds are just used as food additives or extracts (Carvalho *et al*., 2009). Seaweeds are getting importance in various fields ranging from food to medical (Yu-Qing *et al.* 2016).

The present study is the first published data on the proximate composition, vitamins, minerals and heavy metals content of *U. lactuca* collected from Pameungpeuk waters, Indonesia. As an edible seaweed, *U. lactuca* by coastal communities of Pameungpek generally utilised as delicious fresh vegetables. The purpose of this present study is to know the nutritional composition of the edible dried seaweed *U. lactuca* with a view to utilisation in human nutrition.

## MATERIALS AND METHOD

### Sample Collection and Preparation

Seaweed sample was collected from Pameungpeuk waters, Indonesia. Immediately after collection, the seaweed sample was cleaned and washed with seawater to remove sand, debris, epiphytes and other extraneous matter attached to the thalli and transported to the laboratory. In the laboratory, the sample was sorted and then thoroughly cleaned by rinsing with distilled water to remove the surface salty materials. It was air dried with sun directly for five days and later ground in a blender. The powdered samples were subsequently kept in dark container and stored in the room temperature for further analysis.

### Proximate Analysis

The moisture content (%) was determined by drying 2 g *U. lactuca.* The sample was put into an oven at 105°C and heated for 3 h. The dried sample was put into desiccator, allowed to cool and reweighed (AOAC, 1990). Ash content (%) was determined heating *U. lactuca* for 4 in a muffle furnace at 550°C until it turned white and free of carbon. The sample was then removed from the furnace, cooled in a desiccator to a room temperature and reweighed immediately (AOAC, 1990).

Total fat content (%) was determined by loosely wrapping 2 g *U. lactuca* with a filter paper and put into the thimble which was fitted to a clean round bottom flask, which has been cleaned, dried and weighed. The flask contained 120 mL of petroleum ether. The sample was heated with a heating mantle and allowed to reflux for 5 h. The heating then stopped and the thimbles with the spent samples kept and later weighed (AOAC, 2000). Total protein (%) was calculated from the elemental N determination using the nitrogen-protein conversion factor of 6.25 according to the standard AOAC method (2000). The carbohydrate content (%) was estimated by difference: 100 – (moisture + ash + protein + fat) %. The dietary fibre content (%) was determined by weighting 2 g *U. lactuca* and 1 g asbestos and put into 200 mL of 1.25% of H_2_S0_4_ and boiled for 30 min. Solution and content then filter by Buchner funnel and residue was put into 200 mL boiled NaOH. Boiling continued for 30 min, then transferred to the Buchner funnel and filtered. It was then washed twice with alcohol, the material obtained washed twice with petroleum ether. The residue obtained was put in a clean dry crucible and dried in the oven to a constant weight (AOAC, 1999).

### Vitamin, Mineral and Heavy Metal Analysis

Vitamin A content was determined by using HPLC Alliance Waters, Photo Diode Array (PDA) with LiChrospher 100 RP-18 (5um) 4 mm × 250 mm column while vitamin B1 and B2 content were determined by using UPLC H Class Waters, Photo Diode Array (PDA) with ACQUITY UPLC BEH Amide 1.7 um 2.1 × 100 mm column. For the determination of minerals content (calcium, potassium, iron, and sodium) content was determined by the standard AOAC method (2000). Phosphorous content was determined by spectrophotometric method. While the heavy metals (mercury, cadmium, arsenic and lead) content were determined by the standard AOAC method (1980).

## RESULTS

The dried seaweed *Ulva lactuca* from Pameungpeuk waters was studied for proximate, vitamins and minerals analysis. Proximate analysis was done for moisture, ash, protein, fat, carbohydrate and dietary fibre, values of which were found to be 16.9%, 11.2%, 13.6%, 0.19%, 58.1%, 28.4% (dry weight basis) respectively (Table 1). The high protein value but low fat was observed in *U. lactuca.* Carbohydrate and dietary fibre contributed higher value in *U. lactuca.*

Vitamin analysis was done for vitamin A, vitamin B1 and vitamin B2, the values of which were found to be less than 0.5 (IU/100 g), 4.87 mg/Kg, 0.86 mg/Kg respectively (Table 1). Mineral contents are concerned, sodium (Na), calcium (Ca), iron (Fe), potassium (K) and phosphorus (P) were determined in which the values were found to be 364 mg/100 g, 1,824 mg/100 g, 14 mg/100 g, 467 mg/100 g and 0.06 mg/100 g, respectively (Table 1). Heavy metals’ analysis was done for mercury (<0.005 mg/Kg), arsenic (0.09 mg/Kg), cadmium (0.48 mg/Kg) and lead (0.18 mg/Kg) (Table 2).

## DISCUSSION

The moisture, ash, protein, fat, carbohydrate and dietary fibre of *U. lactuca* collected from the Pameungpeuk waters, Indonesia is shown in Table 1. Moisture content is an important criterion in determining the shelf-life and quality of processed seaweed meals as, high moisture may hasten the growth of microorganisms (Rohani-Ghadikolalel *et al.* 2012). In this study, the moisture content was 16.9%. This result was higher than previous study range 0.95%–14.57% (Khairy & El-Shafay 2013) and 10.5% (Abirami & Kowsalya 2011).

Apart from the species specific difference, geographical location and local environmental condition can influence the proximate composition of seaweeds (Rohani-Ghadikolalel *et al.* 2012). In addition, by drying and storage of samples are likely to affect the moisture content of the samples examined. Until now, the National Standardization Agency of Indonesia has not yet set a quality standard of seaweed *U. lactuca.* If compared with other commercial species sold in Indonesia, such as *Eucheuma* sp. (32%,), *Gracilaria* sp. (25%), *Turbinaria* sp. (20%) and *Sargassum* sp. (20%), the moisture obtained in this study is relatively low.

The ash content in *U. lactuca* was examined in this study, 11.2%. This result was higher than reported by Abirami and Kowsalya (2011) was 10.5% and Ortiz *et al.* (2006) was 11%, but lower than reported by Rohani-Ghadikolalel *et al. (*2012) was 12.4%, Khairy and El-Shafay (2013) range between 17.56–24.49% and Abdel-Khaliq *et al.* (2014) was 17.6%.

The protein content in *U. lactuca* was examined in this study, 13.6%. This result was higher than the protein content was reported by Tabarsa *et al. (*2012) in the *U. lactuca* was 10.89% and Abirami and Kowsalya (2011) was 12.9%, but lower than reported by Xiao-Lim *et al.* (2003) was 15.50%, Ortiz *et al.* (2006) was 37.2%, Abirami and Kowsalya (2011) was 12.9%, Khairy and El-Shafay (2013) range between 16.78–17.88%, and Abdel-Khaliq *et al.* (2014) was 17.6%. Burtin (2003) reported that the protein content in green and red seaweeds are generally higher (ranging from 10% to 30%) compared to brown seaweeds (ranging from 5 h to 15%).

The fat content was examined in this study, 0.19%. This result lower than reported by Xiao-Ling *et al.* (2003) was 1.18%, Abirami and Kowsalya (2011) was 1.2% and Abdel-Khaliq *et al.* (2014) was 0.7%. Apparently, the fat content of *U. lactuca* are relatively in low. Based on the result found in this study and the previous reported show that *U. lactuca* can be regarded as an alternative source of a healthy food for human which has high protein but low in fat.

Carbohydrate is the most important component for metabolism as it supplies the energy needed for respiration and other metabolic processes. (Khairy and El-Shafay, 2013). Carbohydrate was the major component in the proximate composition of *U. lactuca* examined in the present study*.* The carbohydrate content was 58.1%. This result is higher than reported by Chakraborty and Santra (2006) for *U. lactuca* was 35.5%, Khairy and El-Shafay (2013) range between 42.09–46.42% and Abdel-Khaliq *et al.* (2014) was 55.6%, but lower than reported by Ortiz *et al.* (2006) for *U. lactuca* was 61.5%) and 64.2% by Abirami and Kowsalya (2011).

The dietary fibre content was examined in this study, 28.4%. This result lower than reported by Ortiz *et al.* (2006) 60.5%. High levels of dietary fibre content in *U. lactuca* may be considered a potential alternative source of dietary fibre. Vitamin A was examined in this study less than 0.5 IU/100 mg while vitamin B1 (thiamine) and vitamin B2 (riboflavin) are 4.87 mg/kg and 0.86 mg/kg respectively.

The mineral composition examined in this study is shown in Table 1. Calcium content was 1828 mg/100 g higher than other minerals. Potassium, sodium, iron and phosphorus content are 467 mg/100 g, 364 mg/100 g, 14 mg/100 g and 0.05 respectively. Calcium and iron content examined in this study higher than reported by Abirami and Kowsalya (2011) was 1094 mg/100 g and 2.3 mg/100 g. But phosphorus content lower than reported by Abirami and Kowsalya (2011 was 86 mg/100 g. The minerals composition and concentration are species and location specific because seaweeds are able to selectively absorb minerals’ from the surrounding seawater and accumulate them in their thalli (Rohani-Ghadikolalel *et al.*, 2012)

The heavy metal content examined in this study is shown in Table 2. Mercury content is less than 0.005 mg/Kg, whereas arsenic, cadmium and lead were in level of 0.09 mg/Kg, 0.48 mg/Kg and 0.18 mg/Kg respectively.

According to Burtin (2003) seaweeds must meet safety regulation in term of toxicological and bacteriological criteria. This regulation, in addition to the potential nutritional properties of seaweeds, allows the food industry to include seaweeds as raw or semi-processed materials in the formulation of seafood products. The quality criteria applied to edible seaweeds sold in Indonesia revealed the standards according to the National Standardization Agency of Indonesia, where the upper limit for arsenic, cadmium and mercury were less than 1 mg/Kg, while the upper limit for lead was less than 1.5 mg/Kg. The heavy metal content reported in this study was within the tolerable based on the qualification criteria for edible seaweeds sold in Indonesia.

## CONCLUSION

The seaweed *U. lactuca* examined in this study has high protein, carbohydrate and dietary fibre content but low in fat. The result of this study shows that *U.lactuca* may be considered to be developed as an alternative source of a healthy food for human in the future.

## Figures and Tables

**Table 1:** Result analysis of nutrient content of *U. lactuca.*

Nutrients content	Results
Moisture (%)	16.9
Ash (%)	11.2
Protein (%)	13.6
Fat (%)	0.19
Carbohydrate (%)	58.1
Dietary fibre (%)	28.4
Vitamin A (IU/100 g)	< 0.5
Vitamin B1 (mg/kg)	4.87
Vitamin B2 mg/kg	0.86
Sodium (mg/100 g)	364
Calcium (mg/100 g)	1828
Iron (mg/100 g)	14.0
Potassium (mg/100 g)	467
Phosphorus (%)	0.05

**Table 2:** Result analysis of heavy metal content of *U. lactuca.*

Heavy metal	Results (mg/Kg)
Mercury	< 0.005
Arsenic	0.09
Cadmium	0.48
Lead	0.18
